# Synthesis, Characterization and Application of Four Novel Electrochromic Materials Employing Nitrotriphenylamine Unit as the Acceptor and Different Thiophene Derivatives as the Donor

**DOI:** 10.3390/polym9050173

**Published:** 2017-05-13

**Authors:** Shuai Li, Guoliang Liu, Xiuping Ju, Yan Zhang, Jinsheng Zhao

**Affiliations:** 1 Shandong Key laboratory of Chemical Energy Storage and Novel Cell Technology, Liaocheng University, Liaocheng 252059, China; lishuaild@163.com (S.L.); zy@lcu.edu.cn (Y.Z.); 2 Research Institute of Petroleum Exploration and Development, Petrochina, Beijing 100083, China; liuguoliangl@petrochina.com.cn; 3 Dongchang College, Liaocheng University, Liaocheng 252059, China; jxp1127@163.com; 4 State Key Laboratory of Heavy Oil Processing, College of Chemical Engineering, China University of Petroleum (East China), Qingdao 266555, China

**Keywords:** nitrotriphenylamine, electrochemistry, thiophene derivatives, electrochromism, spectroelectrochemistry

## Abstract

In this study, four novel donor–acceptor systems, 4-(2,3-dihydrothieno[3,4-b][1,4]dioxin-5-yl)-*N*-(4-(2,3-dihydrothieno[3,4-b][1,4]dioxin-5-yl)phenyl)-*N*-(4-nitrophenyl)aniline (NETPA), 4-(4-methoxythiophen-2-yl)-*N*-(4-(4-methoxythiophen-2-yl)phenyl)-*N*-(4-nitrophenyl)aniline (NMOTPA), 4-(4-methylthiophen-2-yl)-*N*-(4-(4-methylthiophen-2-yl)phenyl)-*N*-(4-nitrophenyl)aniline (NMTPA) and 4-nitro-*N*,*N*-bis(4-(thiophen-2-yl)phenyl)aniline (NTTPA), were successfully synthesized by Stille coupling reaction and electropolymerized to obtain highly stable conducting polymers, PNETPA, PNMOTPA, PNMTPA and PNTTPA, respectively. The polymers were characterized using cyclic voltammetry (CV), step profiling and UV–Vis–NIR spectroscopy. The band gaps (*E*_g_ values) were 1.34, 1.59, 2.26, and 2.34 eV, for PNETPA, PNMOTPA, PNMTPA and PNTTPA, respectively. In addition, electrochromic switching showed that all polymers exhibit outstanding optical contrasts, high coloration efficiencies and fast switching speeds in the near-infrared region (NIR). These properties make the polymers suitable materials for electrochromic applications in NIR region.

## 1. Introduction

The research field of Conducting polymers (CPs) has been developed since the 1970s, and has attracted much attention due to their promising applications in many optic-electronic devices [1,2]. CPs have many advantages such as low cost, portability, easy preparation and compatibility with flexible substrates [3,4,5]. Therefore, they can be used in many fields such as organic solar cells [6], thin film transistor [7], electrochromic materials [8], electroluminescence displays [9], sensors, etc. [10]. Electrochromic materials have shown considerable prospects for the fabrication of a variety of optoelectric devices including e-papers, displays, chameleon camouflage apparatus, etc. [11]. Based on electrochromic materials, more and more products are realizing commercialization, e.g., the intelligent energy saving windows in the building industry and e-papers. The electrochromic polymers with high application values should meet multiple requirements simultaneously, including robust redox stability, fast switching speed, considerable contrast changes in optical transmittance, multichromic changes, etc.

Apart from the polymers based on thiophene [12], pyrrole [13], phenylene, fluorene or carbazole moieties [14,15,16], triphenylamine (TPA) have attracted considerable interest due to their high hole-transport ability, high mobility and low ionization potentials [17,18,19,20]. Some outstanding works have been done on the synthesis and characterization of the TPA-containing polyamide or polyimides, which was prepared by the chemical polycondensation of diacid and diamine [18]. These types of polymers usually have notable electrochromic property and possess some remarkable characteristics including high molecular weight, high thermal stability, good solubility and film-forming ability, lower oxidation potentials, as well as the presence of multiple redox sites which usually leads to multiple colors [19]. However, the preparation of such polymers is very complicated, only high purity monomer can meet to this purpose, and it needs tedious workup to obtain the monomers with high purity [20]. In addition, the response times of such polymers in electrochromic switching are usually longer than other CPs, e.g., polythiophenes, which is an obvious disadvantage for high quality electrochromic materials [20,21].

Thiophene based CPs usually have some merits concerning their utilization as electrochemical or optoelectronic materials, i.e., outstanding redox stability, high polymerizability by electrochemical method, quick switching speed, precise-controlling of the absorption profile (or the color) through structural modification, flexible mechanical performance and multichromic property [22,23,24]. The combination of TPA units and thiophene units within one polymer backbone has been considered and conducted for the development of novel CPs, which presents a very promising strategy for the explorations of high-quality electrochromic materials by fully taking the advantages of their respective merits [25,26,27,28]. TPA can be easily oxidized on the electrode under low oxidation potential, forming the radical cations of TPA (TPA^+^), which tend to undergo dimerization by the coupling reaction between two TPA^+^ in the para position to the nitrogen centers, leading to the production of tetraphenylbenzidine (TPB). However, further oxidation of TBP cannot initiate the polymerization process due to the high stability of its radical cation formed on the electrode [22,23,24].

The introduction of three thiophene derivative units in three para positions to the nitrogen centers led to the formation of new monomers, and the polymerization abilities of the monomers were greatly enhanced since the polymerization site was transferred from the TPA unit to the thiophene units [26,27]. The thiophene capped TPA monomers could be electrochemically polymerized to star-shaped and cross linked polymers with microporous structures. The band gaps of the resultant polymers could be tuned by the selection of the thiophene units with different electron-donating abilities [26,27]. In our recent work, some new thiophene-TPA hybrid monomers have been developed by protecting one of the para positions of the TPA unit by an inactive unit, and capping the other two para positions of the TPA unit with thiophene units [29,30]. The obtained monomers also had excellent polymerization abilities, and the absorption profiles of the electrochemically obtained polymers could be finely tuned by not only the capping units but also the protectively inactive unit. The protectively inactive unit employed by our previous reports included methoxyl unit and cyano unit, which represent a electron-donating unit and a weak electron-withdrawing unit, respectively, and the effects of the introduction of strong electron-withdrawing unit on the triphenylamine unit need to be further studied. When one of the para positions of TPA is protected by an electron-drawing group such as nitro group, the obtained TPA derivative might become an electron-deficient unit. Novel monomers with Donor (D)–Acceptor (A) configuration with high polymerization abilities could be anticipated by capping the electron deficient TPA derivative on two para positions with thiophene derivatives. According to previous reports, the D-A strategy might represent the most effective method for getting low band gap polymers [31,32,33].

Based on the above considerations, four novel monomers were prepared by Stille coupling reaction taking nitrotriphenylamine as the core and thiophene derivatives as the capping agents. Electrochemical polymerization of the four monomers was successfully carried out in the proper electrolyte with low potentials. The spectroelectrochemical and electrochromic properties of the corresponding polymer film were investigated in detail. All films showed two colors with the variation of the applied potentials and exhibited outstanding optical contrasts, high coloration efficiency and fast switching time, which are good for their application in the area of electrochromic polymers.

## 2. Experimental

### 2.1. Materials

Diphenylamine and 4-fluorobenzonitrile were purchased from J & K Co., Ltd. (Beijing, China). *N*,*N*-dimethylformamide (DMF), sodium hydride, *N*-bromosuccinimide (NBS), 2,3-dihydrothieno[3,4-b][1,4]dioxine (EDOT, 98%), 3-methoxythiophene (99%), 3-methylthiophene (99%), thiophene (99%), bis(triphenylphosphine) dichloropalladium (Pd(PPh_3_)_2_Cl_2_), ethanol (EtOH, 99%), acetone, n-butyl lithium (*n*-BuLi, 2.5M) and tributyltin chloride (97%) were all purchased from Aladdin Reagent Co., Limited (Shanghai, China). Acetonitrile, dichloromethane, sodium perchlorate (NaClO_4_), tetrahydrofuran, toluene and chloroform were obtained from Sinopharm Chemical Reagent Co., Ltd. (Shanghai, China). Indium-tin-oxide-coated (ITO) glass (sheet resistance: <10 Ω sq^–1^, obtained form Zhaihai Kaivo Optoelectronic Technology Co., Ltd., (Zhuhai, China) was cleaned thoroughly.

### 2.2. Equipments

The ^1^H NMR and ^13^C NMR spectrum measurements were carried out on a Varian AMX 400 spectrometer (Varian Inc., Santa Clara, CA, USA), and tetramethylsilane (TMS) was used as the internal standard. The electrochemical polymerization and the subsequent redox analyses were carried out in an one-compartment cell with a VERTEX.5A.EIS analyzer (IVIUM, IVIUM Co., Ltd., Amsterdam, The Netherlands), employing a platinum wire with diameter of 0.5 mm as working electrode, a platinum ring as counter electrode, and a silver wire (0.03 V vs. SCE) as pseudo reference electrode. UV-vis-NIR spectra were recorded on a Varian Cary 5000 spectrophotometer. (Varian Inc., Santa Clara, CA, USA) Digital photographs of the polymer films under different potentials were taken using a Nikon D7200 digital camera (Canon Inc., Tokyo, Japan).

### 2.3. Synthesis 

#### 2.3.1. 4-bromo-*N*-(4-bromophenyl)-*N*-(4-nitrophenyl)aniline (NrArineBr_2_)

**Scheme 1:**
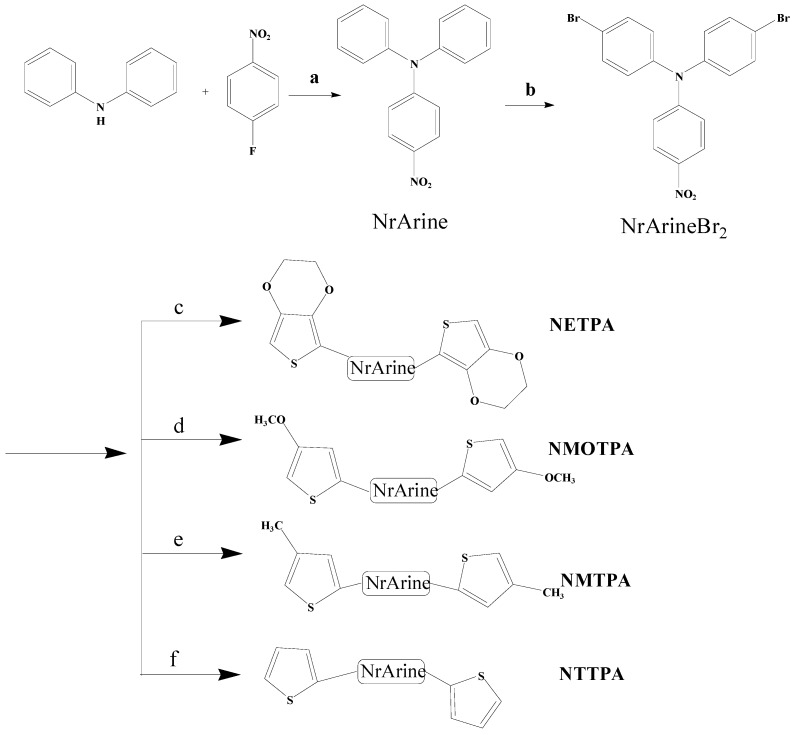
The synthetic route of the monomers: (**a**) NaH, DMF, 140 °С, 15 h, nitrogen atmosphere; (**b**) NBS, DMF, 0 °C, 20 h; and (**c**–**f**) Pd(PPh_3_)_2_Cl_2_, tributyl(2-(3,4-ethylenedioxythiophene))stannane; (**c**) tributyl(2-(4-methoxythiophene))stannane; (**d**) tributyl(2-(4-methylthiophene))stannane; (**e**) tributyl(2-thiophene))stannane; and (**f**) anhydrous THF, reflux, 24 h, argon atmosphere.

The synthetic route of the monomers is shown in Scheme 1. 4-nitro-*N*,*N*-diphenylaniline (NrArine) was synthesized according to previously reported method [32]. The bromination reaction of NrArine was conducted as follows. In a 250 mL round-bottom flask, compound 1 (2.9 g, 10 mmol) was dissolved in 150 mL of DMF, and be stirred magnetically at 0 °C. The above mixture was bubbled with argon gas for 30 min. Then, 4.27 g (24 mmol) of NBS was added to the above solution and is maintained for additional 20 h. After completion, the reaction mixture was poured into 500 mL of the ice water and be stirred mechanically for 0.5 h. Then the precipitant in the mixture was filtered and is washed for three times with water. After drying it in a vacuum drying oven, the resulting solid was purified by recrystallization in anhydrous ethanol. NrArineBr_2_ was obtained as reddish brown solid. ^1^H NMR (CDCl_3_, 400 M Hz, ppm): δ = 8.09 (d, 2H, ArH), 7.50 (d, 4H, ArH), 7.05 (d, 4H, ArH), 6.97 (d, 2H, ArH).

#### 2.3.2. Synthesis of Monomers by Stille Coupling Reaction

The monomers from NETPA to NTTPA as shown in Scheme 1 were synthesized by the Stille coupling reaction. Four thiophene tributylstannane compounds were prepared according to the previously reported methods [33]. The monomer NETPA was synthesized according to the procedures as follows: NrArineBr_2_ (1.8 g, 4 mmol), excess amount of tributyl(2-(3,4-ethylenedioxythiophene))stannane (5.18 g, 12 mmol) and catalytic amount of Pd(PPh_3_)_2_Cl_2_ (0.34 g, 0.48 mmol) was dissolved in anhydrous THF (60 mL) in a 200 mL eggplant flask. Then the flask was evacuated and be filled with argon gas, the procedure was repeated for three times. After that, the mixture was heated to reflux and be stirred magnetically for 24 h in nitrogen atmosphere. Finally, the reaction mixture was cooled down, and the solvent was distilled off under reduced pressure to get a crude solid, which was further purified by column chromatography on silica gel using hexane-DCM mixture solvent as the eluent to give the monomers. Other monomers including NMOTPA, NMTPA and NTTPA were prepared by the same procedure described just above, unless otherwise specified.

NETPA was subjected to column chromatography on silica gel (*n*-hexane-DCM, 2:1, *v*/*v*) to afford the pure substance as a dark red solid, and the yield was 61%. ^1^H NMR (CDCl_3_, 400 M Hz, ppm): 8.05 (d, 2H, ArH), 7.69 (d, 4H, ArH), 7.16 (d, 4H, ArH), 7.01 (d, 2H, ArH), 6.32 (s, 2H), 4.28 (t, 8H). ^13^C NMR (CDCl3, δ, ppm): 152.99, 143.55, 142.23, 140.32, 138.21, 130.60, 127.26, 126.31, 125.46, 118.65, 116.53, 97.86, 64.81, 64.41 (see Supplementary Materials Figure S1).

NMOTPA was purified on silica gel (*n*-hexane-DCM, 3:1, *v*/*v*) to afford an orange powder with 45.7% yield. ^1^H NMR (CDCl_3_, 400 M Hz, ppm): 8.07 (d, 2H, ArH), 7.56 (d, 4H, ArH), 7.18 (d, 4H, ArH), 7.03 (d, 2H, ArH), 6.95 (s, 2H), 6.22 (s, 2H), 3.86 (s, 6H). ^13^C NMR (CDCl3, δ, ppm): 158.70, 152.78, 144.85, 141.73, 140.70, 131.61, 128.09, 126.75, 126.38, 125.53, 119.20, 115.44, 96.36, 57.24 (see Supplementary Materials Figure S2).

The crude product of NMTPA was purified with the same method using the mixture elute (*n*-hexane-DCM, 4:1, *v*/*v*) to afford a red solid with a yield of 63%. ^1^H NMR (CDCl_3_, 400 M Hz, ppm): δ = 8.07 (d, 2H, ArH), 7.58 (d, 4H, ArH), 7.19 (d, 4H, ArH), 7.11 (d, 2H, ArH), 7.05 (s, 2H), 6.88 (s, 2H), 2.30 (s, 6H). ^13^C NMR (CDCl_3_, δ, ppm): 152.92, 144.47, 142.94, 140.57, 138.82, 131.98, 127.05, 126.51, 125.56, 124.81, 120.43, 118.97, 117.93, 15.89 (see Supplementary Materials Figure S3).

The crude product of NTTPA was purified with the same method as that of NMTPA to afford a deep red solid with a yield of 65%. ^1^H NMR (CDCl_3_, 400 M Hz, ppm): 8.08 (d, 2H, ArH), 7.61 (d, 4H, ArH), 7.30 (d, 4H, ArH), 7.19 (m, 4H), 7.08 (m, 4H). ^13^C NMR (CDCl_3_, δ, ppm): 152.88, 144.61, 143.30, 140.63, 131.80, 128.19, 127.31, 126.48, 125.53, 125.06, 123.25, 119.04 (see Supplementary Materials Figure S4).

### 2.4. Electrochemistry

Electrochemical polymerization of the monomers was carried out in a ACN/DCM (1:1, *v*/*v*) solution containing 0.005 M monomer and 0.2 M NaClO_4_ by repetitive cycling at a scan rate of 100 mV s^-1^. The electrodes used in the tests include a platinum wire with diameter of 0.5 mm as working electrode (WE), a platinum ring as counter electrode (CE), and a silver wire as pseudo reference electrode (RE). The WE and CE for cyclic voltammetric experiments were placed 0.5 cm apart during the experiments and all electrodes were cleaned before each experiment. The redox performances and the stability estimates of the polymers were also conducted by the CV method in the monomer-free electrolyte. The Ag wire electrode was calibrated by taking the CV tests of 5 mM of ferrocene (Fc/Fc^+^) in the above electrolyte, and the half-wave potential (*E*_1/2_) is 0.20 V vs. Ag wire [34].

### 2.5. Spectroelectrochemistry and Dynamic Switching Tests

The spectroelectrochemical and the dynamic switching tests were measured with a three-electrode cell, which was constructed in a 1 cm cuvette, where the working electrode was an ITO glass with a surface area of 0.9 × 2.0 cm^2^, the counter electrode was a Pt wire with a diameter of 0.5 mm, and an Ag wire (Ф 0.5 mm) was used as pseudo reference electrode. The polymer films were deposited on the ITO electrode beforehand with a constant polymerization charge of 2.0 × 10^–2 C^. The medium used for the tests is the monomer free electrolyte mentioned above. The potential difference biased on the ITO electrode was actualized by the electrochemical analyzer, and the changes in absorbance intensities were recorded by the spectrophotometer mentioned above.

## 3. Result and Discussion

### 3.1. Electrochemical Polymerization 

**Figure 1:**
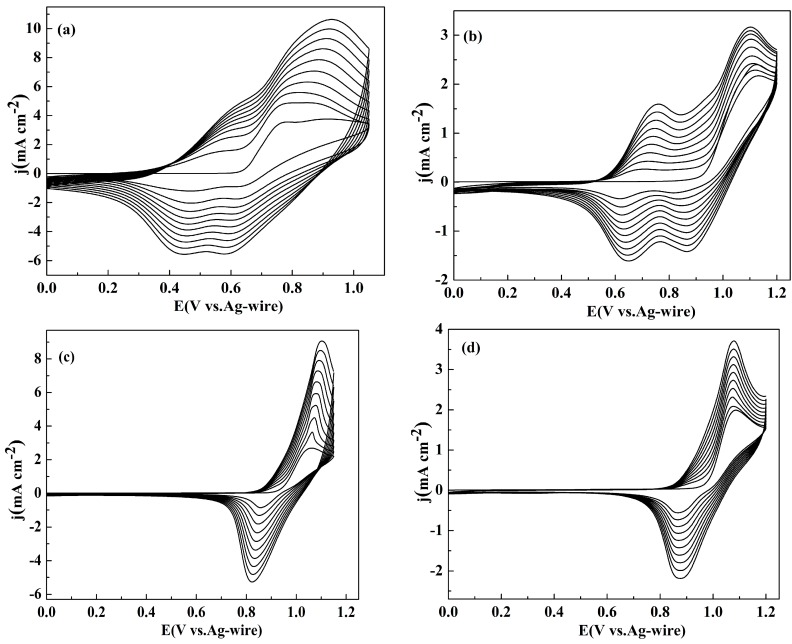
Cyclic voltammogram curves of: NETPA (**a**); NMOTPA (**b**); NMTPA (**c**); and NTTPA (**d**) in 0.2 M NaClO_4_/ACN/DCM solution at a scan rate of 100 mV s^−1^, respectively. *j* denotes the current density. *E* denotes the potential.

All the polymers were deposited on the platinum wire by cyclic voltammogram (CV) with the same potential scan rate (100 mV s^−1^) and the electrochemical polymerization were performed in ACN/DCM (1:1, *v*/*v*) solution containing 0.005 M monomer and 0.2 M NaClO_4_ as a supporting electrolyte. The CV curves of NETPA, NMOTPA, NMTPA and NTTPA were shown in Figure 1. The scanning voltage windows are 0–1.05, 0–1.1, 0–1.15, 0–1.2 V, for NETPA, NMTPA, NMOTPA and NTTPA, respectively. The first cycle of the CV graph was considered as the indication of the redox behavior of the monomer. All the monomers have low polymerization potentials. Figure 1a shows the cyclic voltammograms (CV) of NETPA, which has an onset oxidation potential (*E*_on__set_) at 0.64 V as obtained from the first cycle of the CV graph. No polymer was formed on the WE until the applied potential was higher than 0.9 V, a much higher potential than the *E*_onset_ potential, which suggested that the coupling reaction happened at the α position of the EDOT unit [26,27,28,29]. From the second cycle, a new oxidation peak appeared at 0.47 V, which might arise from the redox activity of the obtained oligomers on the electrode. Subsequently, two couples of redox peaks are available on the CV curves of the NETPA, located at 0.47/0.59, and 0.62/0.83 V, respectively. Similar to that of NETPA, the *E*_on__set_ potential of NMOTPA was 0.92 V, and the potential at which colored materials began to deposit on the WE was 1.1 V. From the second CV cycle, two couples of redox peaks were observed for the CV graph of NMOTPA, located at 0.62/0.69 V, and 0.84/1.14 V, respectively (Figure 1b). The irreversible nature of the second oxidation peaks of both NETPA and NMOTPA were due to the delocalization of spin/charge between the central amine and the terminal thiophene groups, which sets up the possibility of oxidative electrochemical polymerization via coupling at the unsubstituted (α) positions of the thiophene ring [26,27,28,29,30].

As shown in Figure 1c,d, the *E*_on__set_ values for the remaining two monomers were 0.94 and 0.97 V, respectively, for NMTPA and NTTPA. Different with that of NETPA and NMOTPA, the CV curves of NMTPA and NTTPA revealed only one redox peak at 1.1/0.82 and 1.08/0.88 V, respectively, with the oxidation peak directly with the coupling reactions between the α atoms on the thiophene (derivative) ring. Comparing the CV graphs of four monomers, an interesting phenomenon was observed that the widths of the redox peaks of NETPA and NMOTPA were broader than that of NMTPA and NTTPA, which might be due to the higher conjugation lengths of the former monomers than that of the latter monomers, as a resultant of the stronger electron donating abilities of EDOT and 3-methoxythiophene than that of 3-methylthiophene and thiophene as the capping substituent [30]. A common characteristic was also found for the CV of four monomers that, during the sustained CV cycles, the anodic peaks gradually shifted to higher potentials and the cathodic peaks shifted to lower potentials, while the peak currents increased continuously. With the continuous accumulation of polymer on the electrode surface, the resistance of the working electrode increased, and the doping of the anion on the polymer backbone also needed to overcome the additional resistance; all of these might be the cause of the above potential shifts [35].

### 3.2. Electrochemistry Behavior of the Polymer Films

**Figure 2:**
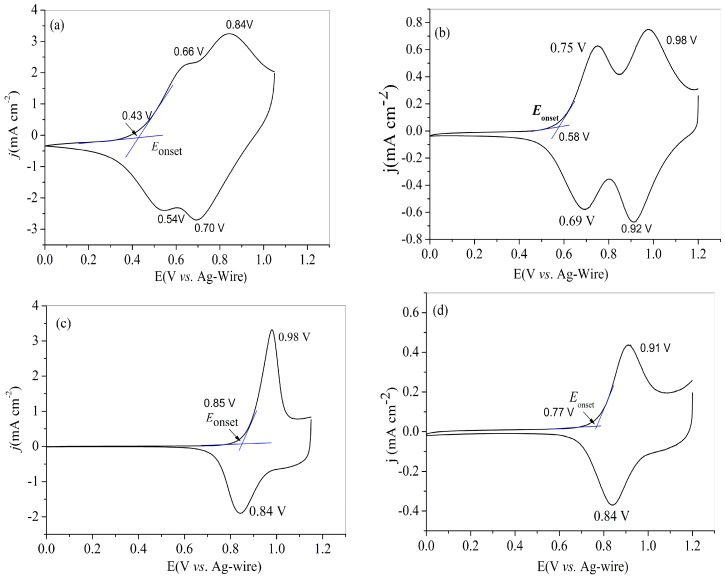
CV curves of: PNETPA (**a**); PNMOTPA (**b**); PNMTPA (**c**); and PNTTPA (**d**) at a determined scan rate of 100 mV s^–1^ in the monomer-free 0.2 M NaClO_4_/ACN/DCM solution.

All of four polymers were prepared beforehand on platinum wires by sweeping the potentials for three cycles. Then, the tests were carried out in monomer-free solution of electrolyte mentioned above between 25 and 300 mV s^−1^. The CV curves of four polymers recorded at the scan rate of 100 mV s^−1^ were used for the identification of the redox peaks. Figure 2a shows the CV curves of PNETPA, two couple of irreversible redox peaks were found at 0.54/0.66 and 0.70/0.84 V, respectively, which are almost fused together to form a continuous and broad peak. Theoretically, the oxidation processes of the nitrotriphenylamine core and the EDOT caps were expected at the same time, but it was very difficult to identify the corresponding redox peaks due to the fusion of the above units in the new conjugation system. As to the redox behavior of PNMOTPA polymer, two reversible redox peaks existed at 0.69/0.75 and 0.92/0.98 V, respectively. However, the PNMTPA film (Figure 2c) and the PNTTPA (Figure 2d) revealed one pair of redox peak at 0.84/0.98 and 0.84/0.91 V, respectively. The *E*_onset_ values of four polymers were recorded at 0.43, 0.58, 0.85 and 0.77 V, (vs. Ag wire), respectively. PNETPA has the lowest *E*_onset_ potential, followed by PNMOTPA, PNTTPA, and PNMTPA. The electron donating strength order of the capping agent was EDOT, 3-methoxythiophene, 3-methylthiophene and thiophene in sequence, which suggested that the strong electron donating abilities of the capping agents are beneficial to reducing the *E*_onset_ values of the monomers. The exception on the relative *E*_onset_ values between PNMTPA and PNTTPA might arise from the greater steric hindrance effect of 3-methylthiophene than that of thiophene [36].

The highest occupied molecular orbital (HOMO) of the obtained polymers could be evaluated from the *E*_onset_ values. Taking the ferrocene/ferrocenium as the standard, its HOMO value is 4.80 V relative to the zero vacuum level, and the HOMO levels of the polymers could be calculated from the equation: *E*_HOMO_ = *E*_onset_ + 4.8 − 0.20 (eV). The potential of the Ag wire electrode was calibrated by taking the CV measurement of 5 mM ferrocene in ACN, and the half-wave potential (*E*_1/2_) was 0.20 V relative to that of the Ag wire reference electrode. The HOMO values of four polymers calculated from the respective *E*_onset_ values were listed in Table 1.

**Figure 3:**
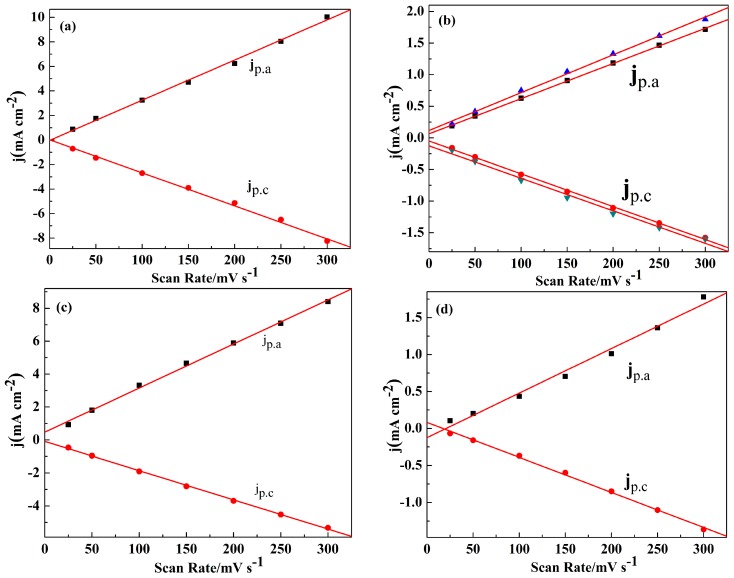
The scan rate dependence of the anodic and cathodic peak current densities graphs of: PNETPA (**a**); PNMOTPA (**b**), there are both two different curves of *j*_p.a_ and *j*_p.c_ since the presence of two couples of redox peaks as shown in Figure 3b; PNMTPA (**c**); and PNTTPA (**d**). *j*_p.a_ and *j*_p.c_ denote the anodic and cathodic peak current densities, respectively.

As shown in Figure S5, the peak current densities (*j*) increased with the increasing of the scan rates between 25 and 300 mV/s in the monomer free electrolyte, indicating that the polymers were deposited on the electrode tightly and uniformly [37]. The scan rate dependence experiments for the four polymer films showed a linear relationship between peak current densities and the scan rates (Figure 3), which demonstrated that the electrochemical processes of the polymer are reversible and not diffusion limited [37].

### 3.3. Electrochemical Stability of the Polymer Films

**Figure 4:**
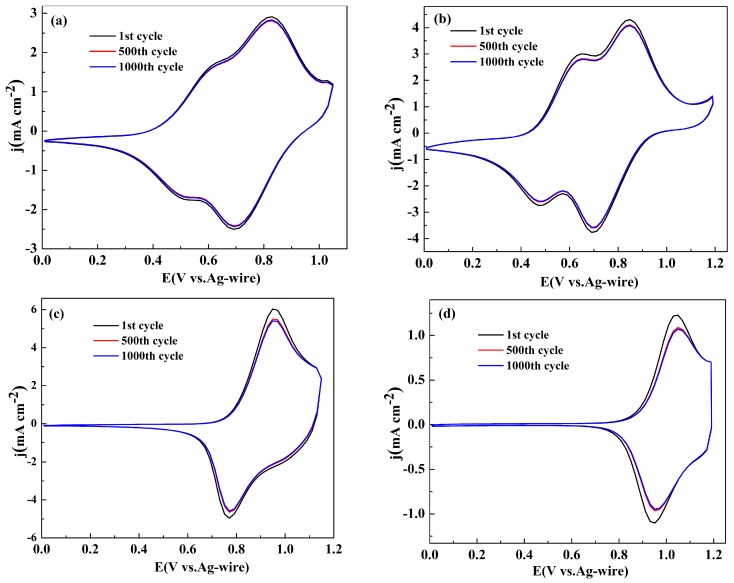
Electrochemical stability of: PNETPA (**a**); PNMOTPA (**b**); PNMTPA (**c**); and PNTTPA (**d**) in the monomer-free electrolyte after 1000 switching cycles by CV.

The long term switching stabilities between the neutral state and the oxidized state were considered as a parameter determining the application perspectives of the polymers in the related fields [37]. To investigate the stabilities of the polymers, the polymer films were prepared on platinum wires by sweeping the potentials for five cycles in the monomer containing electrolyte, and their CV curves were recorded for 1000 cycles at a scan rate of 200 mV s^-1^ in monomer-free electrolyte solution. Figure 4 shows the change in the CV curves for four polymer films between their 1st and 1000th cycles. The total charge consumed in per CV cycle was obtained by integrating the area of the space enclosed by the CV graph. As shown in Figure 4a, the losses of the total charge of the polymers were about 3.1%, 5.5%, 8.3% and 11.3% of their initial values after 1000 cycles, respectively, for PNETPA, PNMOTPA, PNMTPA and PNTTPA. It is convinced that four polymers possessed excellent stabilities, which can meet the requirements of most potential applications. It is obvious that PNETPA has the best stability among four polymers, followed by PNMOTPA, PNMTPA and PNTTPA.

The substituent group on the thiophene unit has a positive effect on the stability of the polymer obtained. The stronger the electron donating ability of the substituent is, the higher the stability of the resulting polymer is. It was supposed that the existence of the polar atom e.g. oxygen atom in the substituent unit is beneficial for the formation of the affinity force between the polymer chains, such as van Edward force, which then promote the stabilities of the resultant polymers. According to previous reports, the degradation process of the polymers could be catalyzed by the oxygen in the air and the trace moisture content in the electrolyte [38]. Considering that the stability test was conducted in the ambient conditions, and stabilities of the polymers would be highly enhanced if the tests were conducted in the conditions excluded with water and oxygen air, a conclusion can be reached that all four polymer films have good switching stabilities between the neutral state and oxidation state for a long time, especially in the case of PNETPA, which make them have important potential applications in the field of electrochromism.

### 3.4. Optical Properties of the Monomers and Films

**Figure 5:**
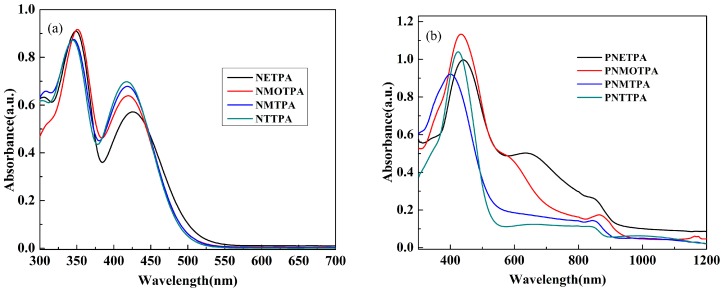
UV–Vis absorption spectra of: the monomers (**a**); and their respective polymers (**b**).

Figure 5 shows the UV-vis absorption spectra of the monomers and their corresponding polymer films deposited on ITO electrode at neural state. All monomers exhibited two characteristic absorption peaks, the high energy absorption band might be due to the nitrotriphenylamine core, and the low energy absorption band might be due to the thiophene derivatives as the capping agent. As shown in Figure 5a, two distinct absorption peaks were observed at 348 and 426 nm for NETPA, 350 and 420 nm for NMOTPA, 346 and 418 nm for NMTPA, and 344 and 416 nm for NTTPA. With the increase of the electron donating abilities of the thiophene derivatives, the absorption peaks of the corresponding monomers has a minor red shift successively. In addition, the optical band gaps (*E*_g_) of the monomers were precisely calculated from the low energy absorption edges (λ_onset_) (*E*_g_ = 1241/λ_onset_). The λ_onset_ values of the polymers are 512, 497, 493 and 489 nm for NETPA, NMOTPA, NMTPA and NTTPA, respectively, and the corresponding *E*_g_ values are calculated to be 2.42, 2.50, 2.52, 2.54 eV, accordingly (see Table 1). It was found out that NETPA has the lowest *E*_g_ value due to the incorporation of the EDOT unit with the strongest electron-donating ability among four thiophene derivatives [36]. Undoubtedly, NTTPA had the highest *E*_g_ value due to the weakest electron-donating ability of thiophene group. In order to get more information about the electronic configuration of the monomers, the DFT calculations were carried out on the B3LYP/6-31G level employing the Gaussian03 program.

**Figure 6:**
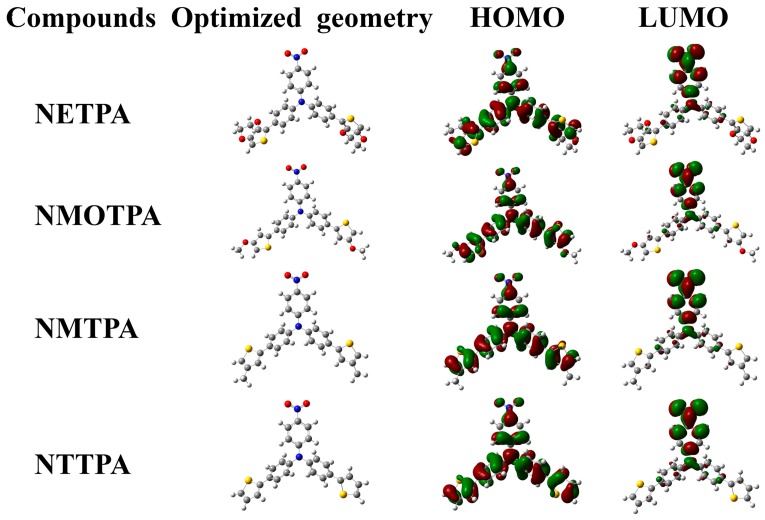
The ground-state electron density distributions of the highest occupied molecular orbital (HOMO) and lowest unoccupied molecular orbital (LUMO).

The ground-state electron density distributions of the HOMO and LUMO orbits are illustrated in Figure 6. As we can see from Figure 6, the HOMO and LUMO orbits of the four monomers largely delocalized on the aromatic rings, which shows fully that all four new synthetic monomers have a large π-conjugated system. In the HOMO orbits, the π electrons mainly distributed on the thiophene derivatives and benzene units directly linked with the thiophene units. However, the π electrons mainly distributed on the nitrobenzene units in the LUMO orbits. By comparing the distribution of electron cloud in different orbits, it is clear that there are intramolecular charge transfers within the monomers reported in this study, which is the typical characteristic of the D-A-D type monomers. The band gap values from DFT calculation were 2.68, 2.75, 2.77, and 2.79 eV, respectively, for NETPA, NMOTPA, NMTPA and NTTPA (see Table 1). Along with the increase in the electron-donating abilities of the thiophene derivatives as the capping agent, the band gap values of the monomer decreased in sequence, which was roughly consistent with the band gap values based on optical experiments. It was noticed that the *E*_g_ values obtained from the theoretical calculation method was about 0.25 eV higher than the value from optical experiments, which might be due to various effects such as solvent effects and other experimental conditions.

Figure 5b shows the UV–Vis absorption spectra of four polymer films deposited on ITO electrode, which was dedoped in the monomer free electrolyte and be washed with acetonitrile for three times. A well defined main peak was observed for all of the polymers, and is located at 440, 434, 400 and 424 nm, respectively, for PNETPA, PNMOTPA, PNMTPA and PNTTPA, which showed a slight redshift compared with that of their corresponding monomers. The redshifts might be an indication of the formation of the long conjugated polymer chains due to the fact that the longer wavelength the absorption peak is, the higher conjugation length the polymer is [30]. Both PNETPA and PNMOTPA polymer films showed shoulder peaks and be located at 634 and 578 nm, respectively, which might be caused by the intramolecular charge transfer along the polymer backbone from the electron-rich thiophene derivative (EDOT or 3-methoxylthiophene) to the electron deficient nitrotriphenylamine unit [31].

The *E*_g_ values of the polymers were calculated to be 1.34, 1.59, 2.26, and 2.34 eV, respectively, for PNETPA, PNMOTPA, PNMTPA and PNTTPA (see Table 1). From PNETPA to PNTTPA, the *E*_g_ values of the polymers increased in sequence, which was probably due to the reduced electron-donating abilities of the thiophene derivatives as the capping agent [35]. The absence of the shoulder peaks in case of both PNMTPA and PNTTPA might due to the weak electron donating abilities of them and their inferior matches with the introtriphenylamine core. For comparison, the performance parameters of similar polymers with the only differences being the protectively inactive units are also listed in Table 1. Unfortunately, the introduction of the nitrotriphenylamine as the linkage unit had no apparent advantages in reducing the bandgaps of the respective polymers when compared with other linkage units such as 4-methoxytriphenylamine and 4-cyanotriphenylamine were employed. The reason might be that the nonplanar and the propeller like molecular configuration of triphenylamine make the electron withdrawing effects of nitro unit cannot be delocalized over the whole molecular, and then nitrotriphenylamine cannot be used as the acceptor unit for the construction of D-A-D type monomer [29,30,31].

### 3.5. Electrochromic Properties of the Polymer Films

**Figure 7:**
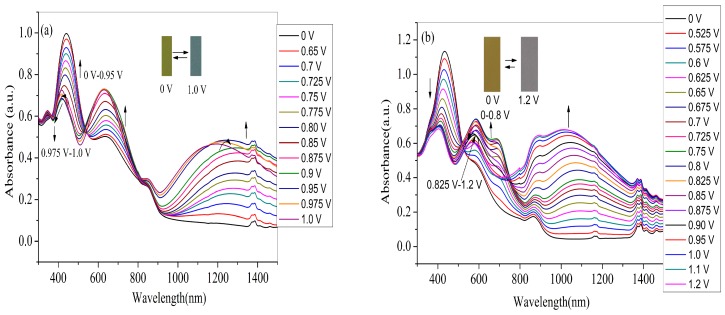

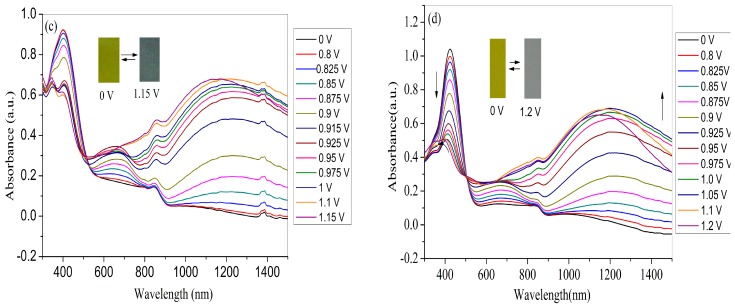
Spectroelectrochemical spectra of polymer films coated on ITO electrode as applied potentials between neutral and oxidized state in monomer-free electrolyte: (**a**) PNETPA; (**b**) PNMOTPA; (**c**) PNMTPA; and (**d**) PNTTPA.

For the application in devices and high-performance displays, the spectroelectrochemical properties of the polymer films should be examined beforehand, which could be manifested by the changes in the optical absorption spectra at various applied voltages. Therefore, four polymer films coated on ITO electrode (with the same polymerization charge of 2.0 × 10^–2^ C) were switched at different potential (between neutral to oxidized state) in monomer-free electrolyte in order to obtain the in-situ UV–Vis–NIR spectra. As shown in Figure 7a, the PNETPA film exhibits two absorption peaks (440 nm and 634 nm) at neutral state. The absorption peak at 440 nm can be ascribed to the π-π* transition. As the applied voltage increased, the intensities of the π–π* transition absorption at 440 nm decreased while the intensities of the absorption bands at approximately 634 nm and 1200 nm increased dramatically upon oxidation, which can attribute to the formation of polaron and bipolarons, respectively [36]. The changes in the absorption spectra were accompanied by color changes from green color at neutral state to steel gray color at the oxidized state.

As shown in Figure 7b, at the neutral state, the PNMOTPA film exhibits pale brown color with an absorption at 434 nm due to the π-π* transition and a shoulder peak centered at 578 nm. Upon oxidation of PNMOTPA film (Figure 7b), the intensity of the transition absorption at 434 nm decreased and two obvious peaks at 600 and 1050 nm occurred corresponding to the polaronic and bipolaronic bands [36]. However, when the applied voltage is larger than 0.975 V, the absorption intensity at about 600 nm decreased gradually, at the same time the bipolaron band was intensified at the expense of the reduction in polaron band [36]. The PNMOTPA film turned into a transmissive grey color at the full doped state (1.2 V) (inset of the Figure 7b).

The PNMTPA film also exhibits an absorption at 400 nm due to the π-π* transition at neutral state (Figure 7c). The π–π* transition peak decreased with the increase of the applied potential and new absorption bands evolved at around 660 nm and 1250 nm during the moderate oxidation of the polymer in the NIR region. The appearance of charge carrier bands could also be attributed to the evolution of polaron and bipolaron bands. Similar to PNMOTPA, there was also a reduction of the absorption band at 660 nm when the potentials are higher than 0.90 V; the reason for which is that the formation of bipolaronic species led to the diminution of polaronic population. The color of PNMTPA changed from yellow green at neutral state to dark gray at the oxidized state (inset of Figure 7c).

For PNTTPA film, it also exhibits an absorption at 424 nm due to the π–π* transition at neutral state (Figure 7d). As the applied voltage increases, the intensity of the π–π* electron transition absorption at 440 nm decreased while the intensity of the absorption bands at approximately 634 nm and 1200 nm increased dramatically upon oxidation, The reason is the same as the former three polymers. The color of PNTTPA changed from bright yellow at 0 V to transmissive grey at 1.2 V (inset of Figure 7d).

Four polymers exhibited different spectroelectrochemical properties and colors due to the different electron-donating abilities of the thiophene derivatives as the capping agent. The possibly existing D-A interactions or intramolecular charge transfers might be accounting for the lower band gaps of PNETPA and PNMOTPA than that of PNMTPA and PNTTPA. An important merit of the present study lies in the facil modulation of the neutral state colors of the polymers by changing the electron donating abilities of the capping agent of the monomers. To further establish the colors of the polymers, the colorimetric properties were characterized using CIE1976 color space (L* a* b*). The values of the relative luminance (*L*), hue (*a*) and saturation (*b*) were measured in the neutral and oxidized states of the polymers and are summarized in Table 2.

### 3.6. Switching Properties of the Polymer Films

**Figure 8:**
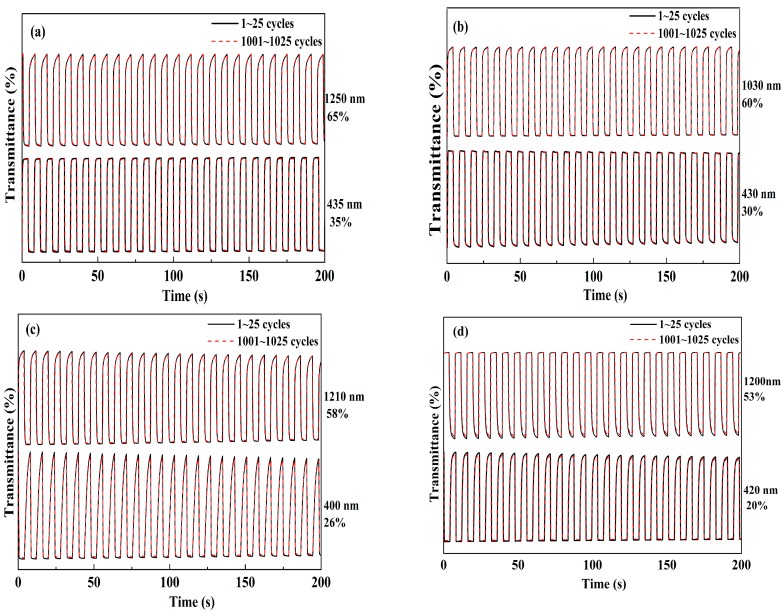
Electrochromic switchings, percent transmittance changes of: PNETPA between 0 and 1.05 V (**a**); PNMOTPA between 0 V and 1.2 V (**b**); PNMTPA between 0 V and 1.15 V (**c**); and PNTTPA between 0 V and 1.2 V (**d**).

The ability for a rapid color change between its neutral and full doped state at given wavelengths is important for the polymers in electrochromic applications. For this purpose, the changes in optical transmittance (Δ*T*%) was monitored as a function of time at their maximum wavelength, and at the same the redox states of the polymer films were regulated by stepping potential periodically between their reduced and oxidized states. The response time and the Δ*T*% value could be obtained directly from the transmittance versus time graphs. Four polymer films were all coated on ITO electrode with the same polymerization charge of 2.0 × 10^−2 C^ and with an identical area of 1 cm^2^. The dynamic studies of the polymers were conducted in a monomer-free solution with a switching interval of 4 s. Figure 8 shows the changes in Δ*T*% values with time at determined wavelength as annotated in the graphs. The Δ*T*% of the PNETPA was calculated to be 35% at 435 nm and 65% at 1250 nm (Figure 8a). The three other polymer films also revealed superior optical contrasts, and the data for PNMOTPA were 30% at 430 nm and 60% at 1050 nm, the data for PNMTPA were 26% at 400 nm and 58% at 1210 nm, and the data were 20% at 420 nm and 53% at 1200 nm for PNTTPA. After 1000 cycles of switches, the losses in percent transmittance contrast values of all four polymer films were slight (all less than 6%, see Table 3), which indicated all of the polymers had dynamically switching stabilities. Compared with the Δ*T*% values in the visible region, all of the polymers had a much higher Δ*T*% values in the near-infrared region, which make the polymers have great application prospects in the preparation of near infrared devices [39].

The response time is defined as the time needed to fulfill the 95% of the full transmittance contrast during the doping or dedoping processes. The response time of PNETPA was found to be 0.7 s at 435 nm and 0.6 s at 1250 nm from the neutral to the oxidized state. PNMOTPA had the switching times of 0.4 and 0.6 s from the neutral to the oxidized state at 430 and 1030 nm, respectively. PNMTPA also had response times of 1.6 and 0.7 s from the neutral to the oxidized state at 400 and 1210 nm, respectively. While, the response time of PNTTPA was found to be 1.8 s at 420 nm and 1.7 s at 1200 nm from the neutral to the oxidized state (Table 3). Comparing the Δ*T*% values and the response times among four polymer films, it was interesting to find that the strong electron-donating abilities of the thiophene derivatives as the capping agents were beneficial for improving the electrochromic properties of the polymers as to the Δ*T*% values and the dynamic switching speeds. The oxygen atoms on the side chains of both NETPA and NMOTPA might render the possibility for the formation of ∙∙∙O∙∙∙O∙∙∙ non-covalent bond between the adjacent molecular segments, a more compact microstructure of the resulting polymers might be found than two oxygen atom free polymers. The compact structure might be beneficial to shorten the travel of electron transport, which may be the reason why these two polymers have a faster response time than that of PNMTPA and PNTTPA [40].

The coloration efficiency (CE, η) is also an important parameter for the evaluation of the electrochromic materials. *CE* can be calculated using the equations given below [30]:

(1) $$\Delta \mathrm{OD}=\log\left(\frac{T_{b}}{T_{c}}\right)$$

(2) $$\eta =\frac{\Delta \mathrm{OD}}{\Delta Q}$$

where *T*_b_ and *T*_c_ are the transmittances before and after coloration, respectively. Δ*OD* is the change of the optical density, which is proportional to the amount of created color centers. η denotes the coloration efficiency (CE). Δ*Q* is the amount of injected/ejected charge per unit sample area. The CE value of PNETPA was calculated to be 322.5 cm^2^ C^−1^ at 435 nm and 358.3 cm^2^ C^−1^ at 1250 nm. The data for PNMOTPA were recorded as 207.8 cm^2^ C^−1^ at 430 nm, 288.6 cm^2^ C^−1^ at 1030 nm. The data for PNMTPA film were 247.3 cm^2^ C^−1^ at 400 nm and 334.6 cm^2^ C^−1^ at 1210 nm. Meanwhile, the data for PNTTPA were 280.3 cm^2^ C^−1^ at 420 nm and 360.6 cm^2^ C^−1^ at 1200 nm (Table 3). From the above results, it could be concluded that all of the polymer films have high coloration efficiencies, which might be beneficial to reduce the energy consumption in the practical applications. In addition, the *CE* values of four polymers are comparable with each other and are not influenced by the electron donating abilities of the capping agent. Especially, four polymer films have excellent electrochromic properties in the NIR region, which was characterized by high Δ*T*% values, fast response speed, high *CE* values and high stability performance, which make four polymers to be promising candidates for NIR device fabrications. Furthermore, all of the polymers could perform the color switches among the primary colors, which also provide the possibilities for the application of these polymers in the field of non-emitting display devices [41,42,43].

## 4. Conclusion

In this paper, four novel monomers were obtained by capping the nitrotriphenylamine core with thiophene derivatives. The capping agents used include EDOT, 3-methoxythiophene, 3-methylthiophene and thiophene, and the obtained monomers were named as NETPA, NMOTPA, NMTPA and NTTPA, respectively. All of the monomers have perfect polymerization, and the corresponding polymers could be facilely deposited on the electrode surface with low voltage potentials by potentiostatic or CV method. The CV performances of the polymers indicated that the *E*_onest_ of the polymers decreased along with the increase in the electron-donating abilities of the capping agents, and the same was found for the *E*_g_ values of the polymers. The absorption bands from the intramolecular charge transfer (ICT) being typical of the D-A type polymers were only found on the polymers of PNETPA and PNMOTPA, which make these two polymers low bandgap polymers. The ICT within PNETPA and PNMOTPA might be promoted by the good matching of the electron-rich capping agent and the electron deficient core unit since the strong electron donating abilities of EDOT and 3-methoxythiophene. The *E*_g_ values of PNMTPA and PNTTPA are much higher than that of the PNETPA and PNMOTPA, which might due to the weak electron donating abilities of their capping agent and the bad matching between the capping agent and the nitrotriphenylamine core. Electrochromic and kinetic studies demonstrated that all polymers had high contrast ratios, high coloration efficiencies, fast response speeds, excellent stabilities and favorable color switches, which make them very promising in the preparation of NIR device and display device.

## Figures and Tables

**Table 1:** The main parameters of the monomers and the respective polymers. The physical meanings of parameters have been explained in the text.

Compounds	*E*_onset_, vs. (Ag-wire) (V)	λ_max_ (nm) /λ_onset_ (nm)	*E_g_* ^a^ (eV)	HOMO ^b^ (eV)	LUMO ^c^ (eV)	Δ*E* ^d^ (eV)	HOMO ^d^ (eV)	LUMO ^d^ (eV)
NETPA	0.64	348,426/512	2.42	−5.24	−2.82	2.68	−5.39	−2.71
NMOTPA	0.92	350,420/497	2.50	−5.52	−3.02	2.75	−5.59	−2.84
NMTPA	0.94	346,418/493	2.52	−5.54	−3.02	2.77	−5.58	−2.81
NTTPA	0.97	344,416/489	2.54	−5.57	−3.03	2.79	−5.65	−2.86
PNETPA	0.42	440,634/926	1.34	−5.02	−3.68	-	-	-
PNMOTPA	0.58	434/780	1.59	−5.18	−3.59	-	-	-
PNMTPA	0.85	400/550	2.26	−5.45	−3.19	-	-	-
PNTTPA	0.77	424/530	2.34	−5.37	−3.03	-	-	-
PMTTPA ^e^	0.18	435/522	2.37	−4.58	−2.21	-	-	-
PMETPA ^e^	0.11	454/533	2.32	−4.51	−1.29	-	-	-
		705/955	1.29	−4.51	−3.22	-	-	-
P1 ^f^	0.60	416/502	2.47	−5.00	−2.53	-	-	-
P2 ^f^	0.20	441/517	2.39	−4.60	−2.21	-	-	-
		642/900	1.37	−4.60	−3.23	-	-	-

^a^ Calculated from the low energy absorption edges (λ_onset_) *E_g_* = 1241/λ_onset_. ^b^ HOMO = −*e*(*E*_onset_ + 4.8 − 0.2). ^c^ LUMO = HOMO + *E_g_*. ^d^ Δ E is calculated based on DFT. ^e^ Data were taken from Ref. [28]. ^f^ Data were taken from Ref. [29]

**Table 2:** The values of *L*, *a*, and *b* for the polymers in neutral and oxidized states.

Polymers	E vs. (Ag wire) (V)	*L*	*a*	*b*	Colors
PNETPA	0	64.30	−4.07	40.65	green
	1.05	64.73	−5.88	1.51	steel gray
PNMOTPA	0	72.69	−0.45	17.42	Pale brown
	1.2	71.18	4.07	−4.95	light grey
PNMTPA	0	76.77	−8.60	47.10	yellow green
	1.15	68.82	−5.88	6.24	dark gray
PNTTPA	0	93.55	−13.12	51.40	bright yellow
	1.2	86.67	−3.17	6.24	light grey

**Table 3:** The optical contrasts (Δ*T*%), response times and coloration efficiencies (CE) of PNETPA, PNMOTPA, PNMTPA and PNTTPA at different wavelengths.

Compounds	λ (nm)	Optical Contrast (Δ*T*%)	Response Time (s)	Coloration Efficiency (CE, cm^2^ C^−1^)	Retained Optical Activity (after 1000 Cycles, Δ*T*% )
PNETPA	435	35.0	0.7	322.5	98.6
	1250	65.0	0.6	358.3	99.0
PNMOTPA	430	30.0	0.4	207.8	97.7
	1030	60.0	0.6	288.6	98.2
PNMTPA	400	26.0	1.6	247.3	95.3
	1210	58.0	0.7	334.6	97.0
PNTTPA	420	20.0	1.8	280.3	94.2
	1200	53.0	1.7	360.6	96.5

## Supplementary Materials

The following are available online at www.mdpi.com/2073-4360/9/5/173/s1, Figure S1: ^1^H NMR spectrum of NETPA in CDCl_3_ (a). Solvent peak is at δ = 7.26 ppm. ^13^C NMR spectrum of NETPA in CDCl_3_ (b). Solvent peak is at δ = 77.3 ppm; Figure S2: ^1^H NMR spectrum (a) and ^13^C NMR spectrum (b) of NMOTPA in CDCl_3_. The solvent peak in ^1^H NMR is at δ = 7.26 ppm, and the solvent peak in ^13^C NMR spectrum is at δ = 77.3 ppm; Figure S3: ^1^H NMR spectrum (a) and ^13^C NMR spectrum (b) of NMTPA in CDCl_3_. The solvent peak in ^1^H NMR spectrum is at δ = 7.26 ppm and the solvent peak in ^13^C NMR spectrum peak is at δ = 77.3 ppm; Figure S4: ^1^H NMR spectrum (a) and ^13^C NMR spectrum (b) of NTTPA in CDCl_3_. The solvent peak for ^1^H NMR spectrum is at δ = 7.26 ppm. The solvent peak in ^1^H NMR spectrum is at δ = 7.26 ppm and the solvent peak in ^13^C NMR spectrum peak is at δ = 77.3 ppm; Figure S5: CV curves of the NETPA (a), NMOTPA (b), NMTPA (c) and NTTPA (d) film at different scan rates between 25 mV s^−1^and 300 mV s^−1 in the monomer-free^ 0.2 M NaClO_4_/ACN/DCM solution.
