# Supplementary material for: Synthesis, Characterization and Application of Four Novel Electrochromic Materials Employing Nitrotriphenylamine Unit as the Acceptor and Different Thiophene Derivatives as the Donor
Source: Polymers (Basel). 2017 May 13;9(5):173. doi: 10.3390/polym9050173 (PMC6432490; doi:10.3390/polym9050173)
Supplement: Supplementary file 1 [file polymers-09-00173-s001.pdf]

Supporting Information: Synthesis, characterization and application of four novel electrochromic materials employing nitrotriphenylamine unit as the acceptor and different thiophene derivatives as the donor

Shuai Li[1], Guoliang Liu[2], Xiuping Ju[3], Yan Zhang[1], Jinsheng Zhao[1,4*]

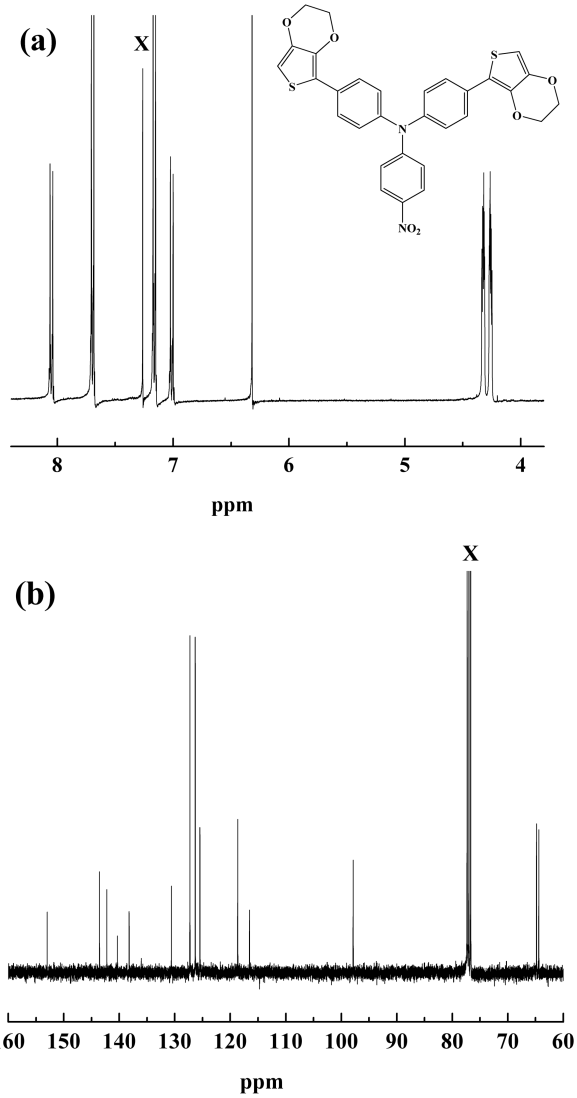

Fig.S1. ^{1}H NMR spectrum of **NETPA** in $CDCl_3$ (a). Solvent peak is at δ=7.26 ppm. ^{13}C NMR spectrum of **NETPA** in $CDCl_3$ (b). Solvent peak is at δ = 77.3 ppm.

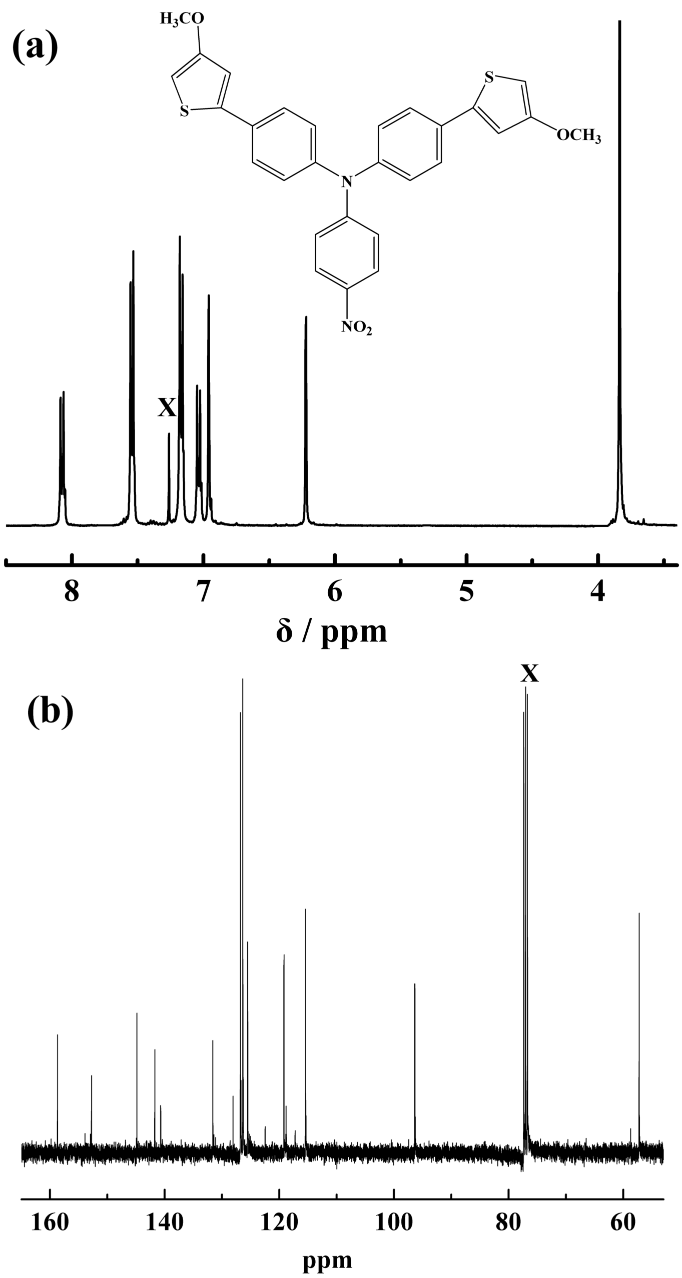

Fig.S2. ^{1}H NMR spectrum (a) and ^{13}C NMR spectrum (b) of **NMOTPA** in $CDCl_3$. The solvent peak in ^{1}H NMR is at δ = 7.26 ppm, and the solvent peak in ^{13}C NMR spectrum is at δ = 77.3 ppm.

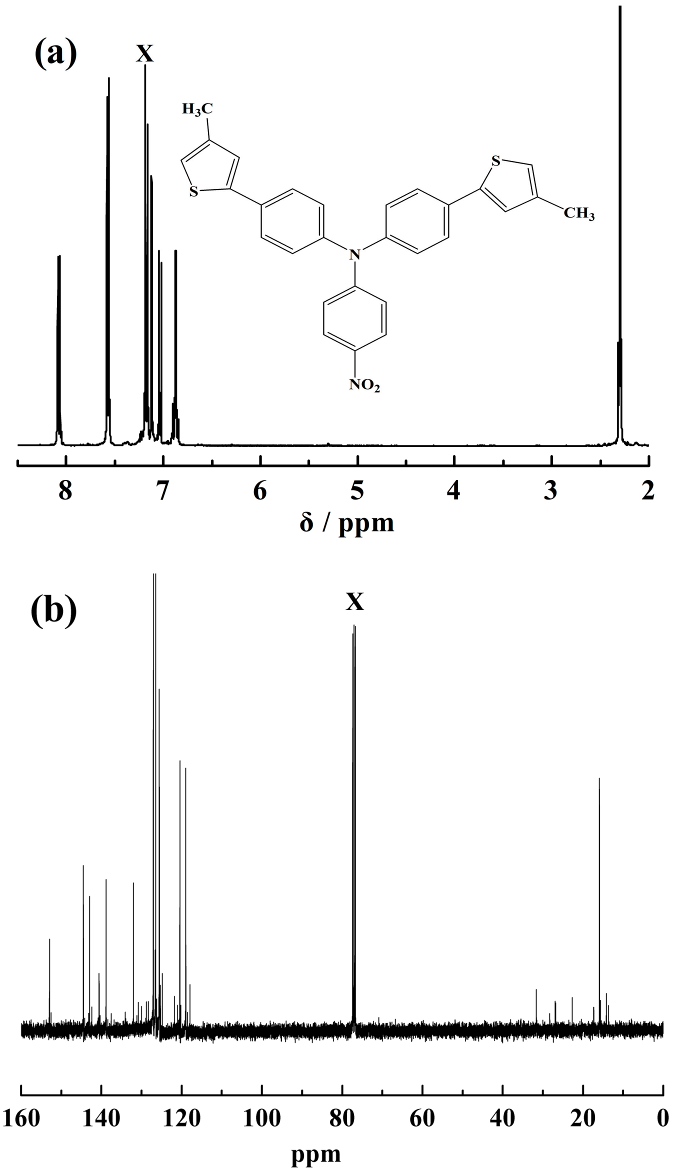

Fig.S3. ^{1}H NMR spectrum (a) and ^{13}C NMR spectrum (b) of **NMTPA** in $CDCl_3$. The solvent peak in ^{1}H NMR spectrum is at δ = 7.26 ppm and the solvent peak in ^{13}C NMR spectrum peak is at δ = 77.3 ppm.

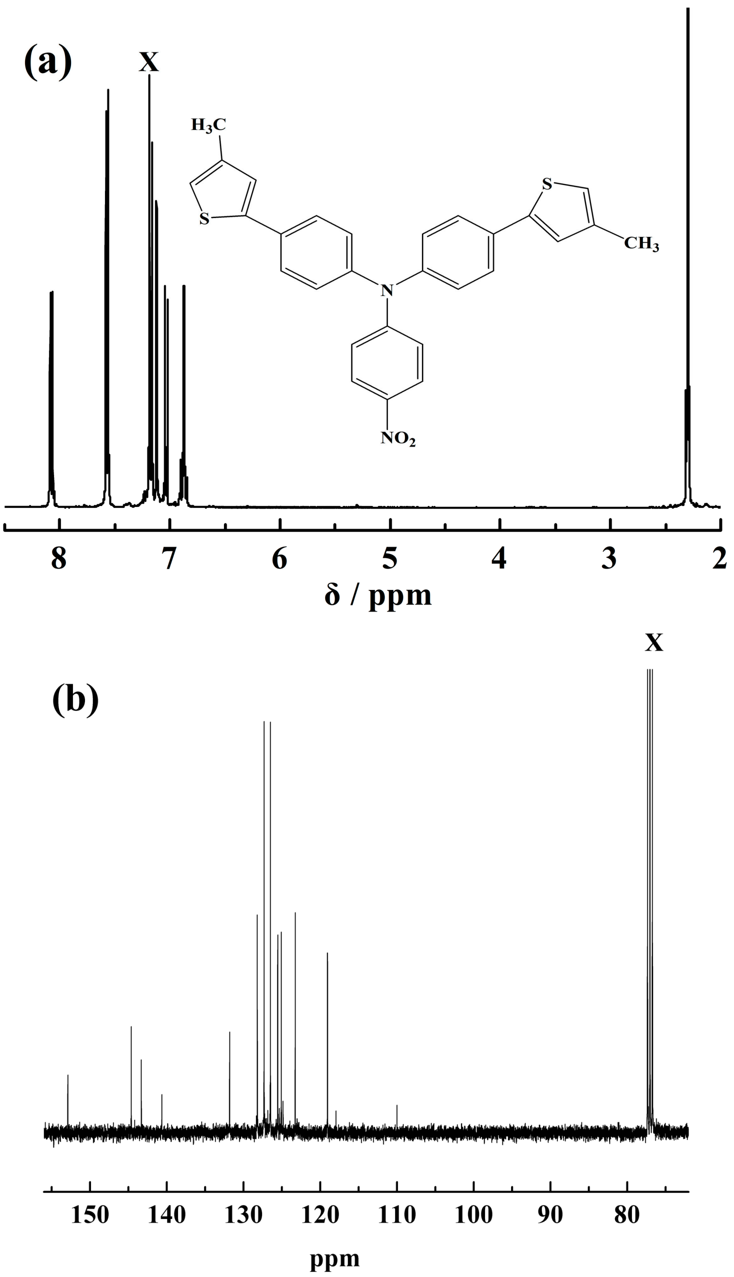

Fig. S4. ^{1}H NMR spectrum(a) and ^{13}C NMR spectrum (b) of **NTTPA** in $CDCl_3$. The solvent peak for ^{1}H NMR spectrum is at δ = 7.26 ppm. The solvent peak in ^{1}H NMR spectrum is at δ = 7.26 ppm and the solvent peak in ^{13}C NMR spectrum peak is at δ = 77.3 ppm.

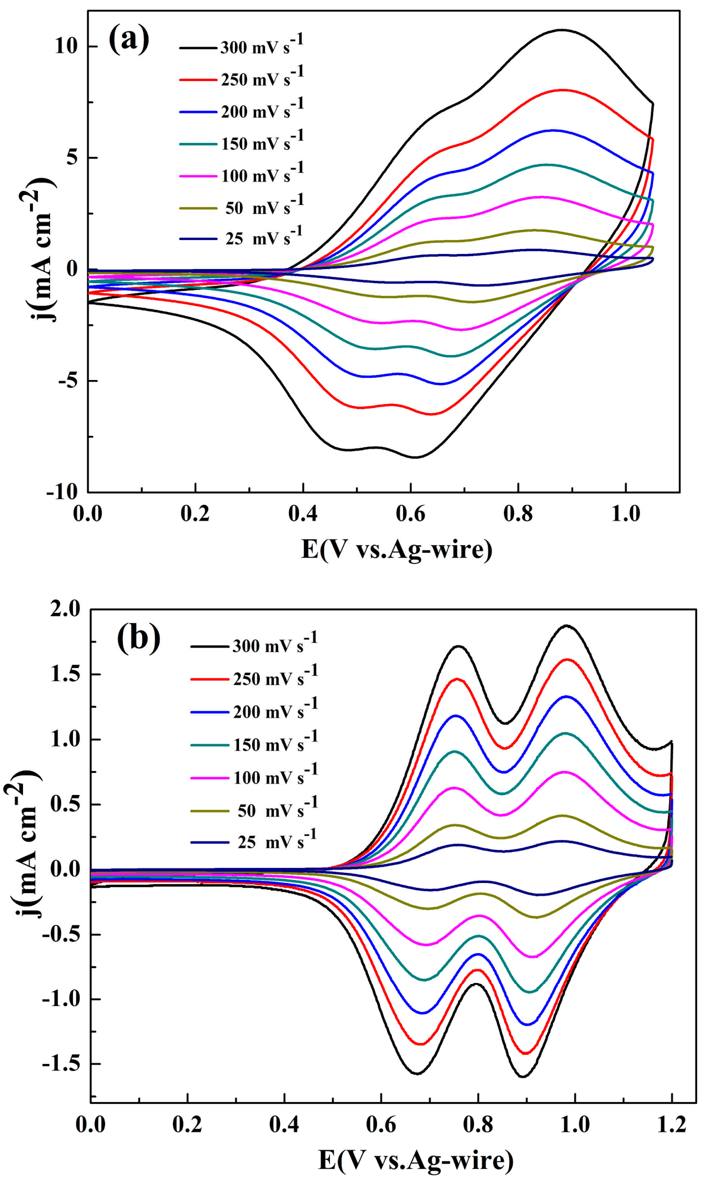

Fig. S5. CV curves of the NETPA (a), NMOTPA(b), NMTPA (c) and NTTPA (d) film at different scan rates between 25 mV $s^{-1}$and 300 mV s^{-1} in the monomer-free 0.2 M $NaClO_4$/ACN/DCM solution.
